# African Glucose-6-Phosphate Dehydrogenase Alleles Associated with Protection from Severe Malaria in Heterozygous Females in Tanzania

**DOI:** 10.1371/journal.pgen.1004960

**Published:** 2015-02-11

**Authors:** Alphaxard Manjurano, Nuno Sepulveda, Behzad Nadjm, George Mtove, Hannah Wangai, Caroline Maxwell, Raimos Olomi, Hugh Reyburn, Eleanor M. Riley, Christopher J. Drakeley, Taane G. Clark, MalariaGEN Consortium

**Affiliations:** 1 Joint Malaria Programme, Kilimanjaro Christian Medical College, Moshi, Tanzania; 2 Department of Infection and Immunology, Faculty of Infectious and Tropical Diseases, London School of Hygiene and Tropical Medicine, London, United Kingdom; 3 Department of Clinical Research, Faculty of Infectious & Tropical Diseases, London School of Hygiene & Tropical Medicine, London, United Kingdom; 4 Pathogen Molecular Biology Department, Faculty of Infectious and Tropical Diseases, London School of Hygiene and Tropical Medicine, London, United Kingdom; 5 Department of Infectious Disease Epidemiology, Faculty of Epidemiology and Population Health, London School of Hygiene and Tropical Medicine, London, United Kingdom; 6 Wellcome Trust Centre for Human Genetics, University of Oxford, Oxford, United Kingdom; Ospedale San Pietro Fatebenefratelli, ITALY

## Abstract

X-linked Glucose-6-phosphate dehydrogenase (G6PD) A- deficiency is prevalent in sub-Saharan Africa populations, and has been associated with protection from severe malaria. Whether females and/or males are protected by G6PD deficiency is uncertain, due in part to G6PD and malaria phenotypic complexity and misclassification. Almost all large association studies have genotyped a limited number of *G6PD* SNPs (e.g. G6PD202 / G6PD376), and this approach has been too blunt to capture the complete epidemiological picture. Here we have identified 68 G6PD polymorphisms and analysed 29 of these (i.e. those with a minor allele frequency greater than 1%) in 983 severe malaria cases and controls in Tanzania. We establish, across a number of SNPs including G6PD376, that only female heterozygotes are protected from severe malaria. Haplotype analysis reveals the *G6PD* locus to be under balancing selection, suggesting a mechanism of protection relying on alleles at modest frequency and avoiding fixation, where protection provided by G6PD deficiency against severe malaria is offset by increased risk of life-threatening complications. Our study also demonstrates that the much-needed large-scale studies of severe malaria and G6PD enzymatic function across African populations require the identification and analysis of the full repertoire of G6PD genetic markers.

## Author Summary

Glucose-6-phosphate dehydrogenase (G6PD) is an essential enzyme that protects red blood cells from oxidative damage. Numerous genetic variants of *G6PD*, residing in the X chromosome, are found among African populations: mutations causing A- deficiency can lead to serious clinical outcomes (including hemolytic anemia) but also confer protection against severe malaria. Epidemiological studies have used some of the genetic markers that cause A- deficiency to establish who is protected from severe malaria, with differing results. Whether females, with one or two copies of mutant genes, males with one copy, or both genders are protected is uncertain. This uncertainty is due to G6PD and malaria phenotypic complexity and misclassification, and to genetic differences between populations and the limited numbers of genetic markers (usually 2) considered. In this study we analysed more than 30 G6PD genetic markers in 506 Tanzanian children with severe malaria and 477 without malaria. We found that only females with one normal and one mutant copy of the gene (heterozygotes) were protected from severe malaria. Further, we established that the G6PD gene is under evolutionary pressure with the likely mechanism being selection by malaria. Our work demonstrates that studies of severe malaria and G6PD enzymatic function across African populations require, in addition to complete and accurate G6PD phenotypic classification, the identification and analysis of the full repertoire of G6PD genetic markers.

## Introduction

Amongst the approximately 190 genetic variants causing clinical deficiency of Glucose-6-phosphate dehydrogenase (G6PD) that have been characterised [1], the A- deficiency is the most common in sub-Saharan Africa populations, and is associated with protection from severe malaria [2,3]. An understanding of how this protection works may assist with the design of anti-malarial vaccines and drugs. Establishing whether malaria patients are G6PD deficient is also important because of the potential use of 8-aminoquinoline drugs (e g, primaquine and its derivatives) for malaria elimination in sub-Saharan Africa [4]. Primaquine is active against all liver stages of Plasmodium, and also offers activity against *P*. *falciparum* gametocytes, thereby blocking transmission to mosquitoes [4]. However, primaquine is haemotoxic, and can cause haemolytic anaemia in G6PD-deficient individuals. G6PD status can be quantified using enzymatic activity assays and is required for unambiguous identification of G6PD-deficiency, especially in mosaic female heterozygotes due to the X-linkage of the trait [5]. Cytochemical methods have been suggested as an alternative [5], but are not efficient for large studies, and genotyping has been used as a high throughput approach. Whilst genotyping approaches have been advocated, there is evidence of extensive diversity at the *G6PD* locus (X chromosome, 16.2kb), with more than 150 single nucleotide polymorphisms (SNPs) reported [1]. Many of these known genetic variants result in amino acid changes and have been detected through sequencing the *G6PD* gene locus in enzyme deficient individuals. The *G6PD* and the Inhibitor of kappa light polypeptide gene (*IKBKG*, involved in immunity, inflammation and cell survival pathways [6], and with mutations linked to Incontinentia Pigmenti [7]) loci overlap each other, including a shared conserved promoter region that has bidirectional housekeeping activity [7]. The region containing the *G6PD* gene and the 5-prime end of the *IKBKG* gene contains Alu elements [7]. The genetic variability in *G6PD* and *IKBKG* is complex [7], and new alleles are still being discovered, making a simple G6PD genetic approach unreliable [8,9].

Despite these limitations, genotyping of the 202A/376G G6PD A-allele (with ∼12% of normal enzymatic activity [10]) has been used extensively in epidemiological studies to investigate protection against severe malaria [8, 10–19]. It has been shown that coexistence of the two mutations is responsible for enzyme deficiency in G6PD A- because they act synergistically in causing instability of the enzyme [20]. They also lead to structural changes in the enzyme protein. However, even in large well-powered studies, associations between 202A/376G G6PD and protection from severe disease have been inconsistent, revealing protective effects in female heterozygotes [8, 11,17,18,19], in male hemizygotes [12,13], in both [14], or no protection [15]. These phenotype-genotype inconsistencies may be explained in part by variation in study design, G6PD and malaria phenotypic complexity and misclassification and incomplete experimental data [8]. However, it has been recognised that allelic heterogeneity, specifically other unknown polymorphisms, has a role [3,5,8], with evidence from studies in West Africa [5,8] for A- deficiency and in Southeast Asia and Oceania for other deficiency types [3]. In particular, in the West African setting, the frequency of the 202A allele is often substantially lower than rates of enzyme deficiency indicating a role for other alleles; inclusion of other *G6PD* polymorphisms (Santamaria 542T/376G—∼2% residual enzymatic activity, Betica-Selma 968C/376G—∼11% activity)[10, 16] was required to capture an association between G6PD deficiency and severe malaria in The Gambia [8].

Further understanding is required of the true extent of genetic diversity within the *G6PD* locus, how this relates to enzyme function, and how it varies between regions and ethnic groups, if genetic epidemiological studies are to provide robust and reproducible findings. A recent study in Mali using 58 SNPs across the *G6PD* gene found differences in core haplotypes and their frequencies between Dogon and Fulani ethnic groups [9]. The latter group is known to have substantially reduced susceptibility to malaria when compared to sympatric populations [9]. Whilst some ethnicity specific SNP associations were observed with mild malaria, the prevalence of severe malaria was too low for any robust associations to be detected.

Here we investigate associations between 68 SNPs within the *G6PD* and surrounding loci (*IKBKG* and *CTAG1A/B*), including the 202, 376, 542, 680 and 968 A- deficiency polymorphisms (referred to here as G6PD202, G6PD376, and so forth), and severe malaria. The work is set within a case-control study (n = 983; 506 cases and 477 controls) conducted in an area of intense malaria transmission in the Tanga region in northeastern Tanzania [17]. To complement the case-control collection, we genotyped samples from 60 healthy parental and child trios (120 parents, 60 children), collected in the same geographical region. We find very strong associations between multiple SNPs across the *G6PD* gene and protection from severe malaria in female heterozygotes but not in hemizygous males. Very high linkage disequilibrium across this locus allowed us to distil this SNP diversity into just 4 G6PD alleles, ranging in frequency from ∼6% to >60%, and 8 common genotypes (>1%), 2 of which are associated with protection from severe malaria.

In summary, this study identifies specific *G6PD* alleles that confer resistance to severe malaria in this population and reveals a potentially important role of female heterozygotes in maintaining the high frequency of *G6PD* polymorphisms in malaria endemic populations.

## Results

Of the severe malaria cases (n = 506), many had severe malarial anaemia (48.6%) or acidosis (57.5%) phenotypes (Table 1). Compared to controls (n = 477), malaria cases tended to be younger and male, and with more individuals outside the 7 main ethnic groups (P<0.05). Malaria cases were less likely to be of blood group O (O vs. A/B/AB, OR 0.726, 95% CI 0.534, 0.986; P = 0.04), with alpha thalassaemia of α-/α- (α-/α- vs. αα/αα or αα/α-, Odds Ratio (OR) 0.639, 95% CI 0.401–1.018, P = 0.06) or present with the sickle cell protective AS genotype (AS vs. other, OR 0.053, 95% CI 0.021–0.132). The sickle cell AS genotype frequency in parents (6.3%) and children (5.4%) in the trio validation study lay between the estimates for the cases (1.0%) and controls (16.5%). As expected, the G6PD542, 680 and 968 polymorphisms found in West African populations [8,9] were all monomorphic in both cases and controls, as well as in the 60 parental-child trios, and were therefore excluded from further analysis.

**Table 1:** Baseline and clinical characteristics.

	Controls (n = 477)		Cases (n = 506)		Difference P-value
Age* (median, range)	(2.9)	(0.9–10.9)	(1.7)	(0.2–10.0)	<0.0001
**Gender—Female**	255	53.5%	236	46.6%	0.04
**Ethnicity**					0.0005
Mzigua	158	33.1%	149	29.4%	
Wasambaa	130	27.3%	130	25.7%	
Wabondei	78	16.4%	79	15.6%	
Chagga	50	10.5%	47	9.3%	
Mmbena	23	4.8%	25	4.9%	
Other	5	1.0%	39	7.7%	
Mngoni	18	3.8%	19	3.8%	
Pare	15	3.1%	18	3.6%	
**Blood group**					0.22
A	115	24.4%	145	29.8%	
AB	20	4.2%	18	3.7%	
B	100	21.2%	107	22.0%	
O	236	50.1%	216	44.4%	
Sickle HbS (rs334)**					<0.0001
AA	385	83.5%	473	97.9%	
AS	76	16.5%	5	1.0%	
SS	0	0.0%	5	1.0%	
**Alpha thalassaemia**					0.15
αα/αα	224	47.2%	236	51.9%	
αα/α-	199	41.9%	184	40.4%	
α-/α-	52	10.9%	35	7.7%	
G6PD202A	92	20.0%	79	16.3%	0.02
G6PD376G	178	38.5%	173	37.4%	0.007
G6PD542T	477	0%	506	0%	-
G6PD680T	477	0%	506	0%	-
G6PD968C	477	0%	506	0%	-
**Clinical phenotype**					NA
Any severe malaria	-	-	506	100%	
Any SMA***	-	-	246	48.6%	
Any CM	-	-	99	19.6%	
Both SMA+CM	-	-	41	8.1%	
Any RD	-	-	146	28.9%	
Acidosis****	-	-	291	57.5%	

* in months, SMA: severe malarial anaemia, CM: cerebral malaria, RD: respiratory distress

**Absence of HbC and HbE mutations

*** hb<5gdl

**** Blood lactate>5mmol/l.

**Fig 1:**
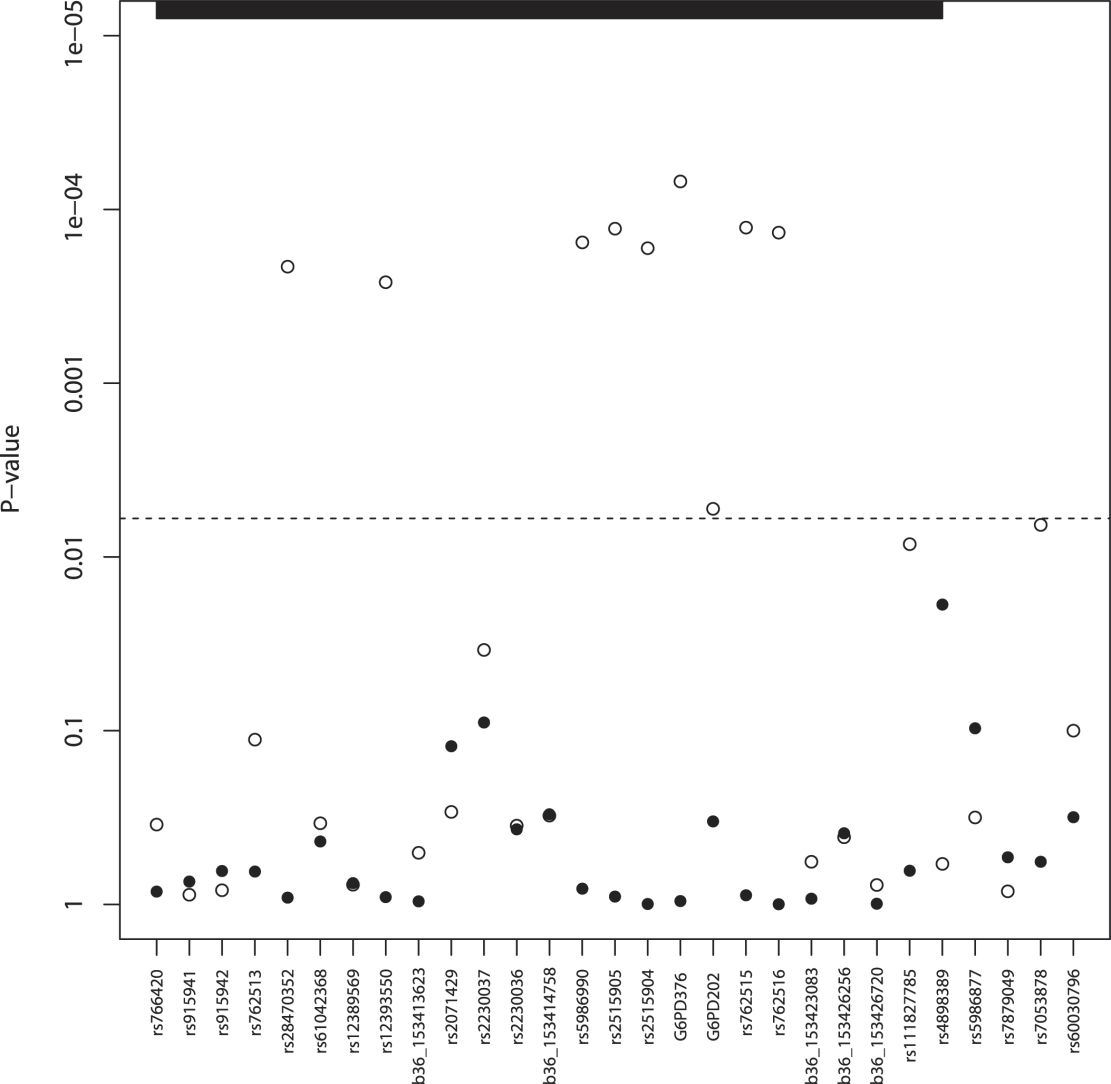
Severe malaria association results* (males—solid, females—hollow circles). * Minimum p-values from single SNP analysis adjusted for age and ethnicity. The dashed line represents a p-value cut-off of 0.006; the vertical dashed line represents a p-value cut-off of 0.006; the *G6PD/IKBKG* region is bolded on the right, and the 5 remaining SNPs are in the *CTAG1A/B* region.

The G6PD202A and G6PD376G A- alleles were among the 29 SNPs retained with minor allele frequency (MAF) in excess of 1% (S2 Table). Both G6PD202A (case 16.3% vs. control 20.0%) and G6PD376G (37.4% vs. 38.5%) allele frequencies were lower in malaria cases than in controls (P<0.02) (Table 1), and broadly similar to the trio study parents (202A 16.8%, 376G 31.3%) and children (202A 15.0%, 376G 24.1%) (S3 Table). A SNP-by-SNP association analysis revealed 11 multiple loci where female heterozygotes appeared to be protected from severe malaria in all its clinical phenotypes (Table 2, Fig. 1, S1 Fig.) except for cerebral malaria where although there was evidence of heterozygous advantage effects (OR ∼ 0.5), they were non-significant due to the small number of cases (99) (P>0.018). The G6PD376 and rs762515 polymorphisms (both flanking G6PD202) were the only SNPs associated with all non-cerebral malaria clinical phenotypes. The association hits across clinical phenotypes included a “core” region consisting of 7 SNPs (rs5986990, rs2515905, rs2515904, G6PD376, G6PD202, rs762515, rs762516) in perfect linkage disequilibrium (D’ = 1), where female heterozygotes were 48.2% and 72.4% less likely to be a severe malaria case (any definition) than female homozygote genotypes (P<0.006, Table 2). By comparison, there were no significant associations between G6PD genotype and severe malaria in hemizygous males (P>0.310).

**Table 2:** G6PD polymorphisms and associations. *

Pheno	SNP	Position	Major/Minor	Female Control	Female Case	Female Cont.	Female OR	Female LCL	Female UCL	Female P	Male Control	Male Case	Male Cont.	Male OR	Male LCL	Male UCL	Male P
SM	G6PD376	153763492	A/G	0.397	0.340	Het	0.436	0.288	0.660	0.00007	0.371	0.367	G v A	1.012	0.672	1.522	0.956
SM	rs762515	153764528	T/C	0.396	0.333	Het	0.455	0.303	0.684	0.00013	0.365	0.364	C v T	1.029	0.690	1.535	0.888
SM	rs2515905	153762075	G/A	0.269	0.221	Het	0.437	0.284	0.672	0.00013	0.241	0.222	A v G	0.972	0.619	1.526	0.901
SM	rs762516	153764663	C/T	0.269	0.217	Het	0.436	0.282	0.672	0.00014	0.239	0.227	T v C	1.000	0.637	1.568	0.999
SM	rs5986990	153761628	G/A	0.395	0.335	Het	0.460	0.306	0.691	0.00015	0.367	0.370	A v G	1.050	0.704	1.566	0.812
SM	rs2515904	153762771	G/C	0.268	0.221	Het	0.441	0.287	0.680	0.00017	0.233	0.222	C v G	1.002	0.636	1.577	0.995
SM	rs28470352	153753490	T/A	0.394	0.334	Het	0.465	0.309	0.701	0.00021	0.369	0.366	A v T	1.022	0.685	1.524	0.914
SM	rs12393550	153758660	G/A	0.387	0.333	Het	0.472	0.314	0.709	0.00026	0.352	0.342	A v G	0.976	0.648	1.471	0.909
SM	G6PD202	153764217	G/A	0.205	0.174	Het	0.518	0.325	0.827	0.00529	0.193	0.147	A v G	0.774	0.461	1.300	0.333
SMA	G6PD376	153763492	A/G	0.397	0.295	Het	0.276	0.153	0.499	0.000008	0.371	0.392	G v A	1.071	0.652	1.757	0.787
SMA	rs762515	153764528	T/C	0.396	0.292	Het	0.292	0.163	0.522	0.000015	0.365	0.397	C v T	1.122	0.692	1.820	0.640
SMA	rs12393550	153758660	G/A	0.387	0.289	Het	0.302	0.169	0.539	0.000025	0.352	0.383	A v G	1.071	0.652	1.759	0.786
SMA	rs5986990	153761628	G/A	0.395	0.297	Het	0.308	0.173	0.549	0.000034	0.367	0.400	A v G	1.123	0.692	1.822	0.639
SMA	rs28470352	153753490	T/A	0.394	0.297	Het	0.317	0.178	0.565	0.000054	0.369	0.400	A v T	1.120	0.691	1.817	0.645
SMA	rs762516	153764663	C/T	0.269	0.163	Het	0.303	0.160	0.576	0.000116	0.239	0.243	T v C	1.058	0.617	1.813	0.838
SMA	rs2515905	153762075	G/A	0.269	0.181	Het	0.320	0.171	0.599	0.000177	0.241	0.245	A v G	1.071	0.626	1.834	0.802
SMA	rs2515904	153762771	G/C	0.268	0.181	Het	0.319	0.170	0.598	0.000179	0.233	0.245	C v G	1.104	0.643	1.895	0.719
SMA	rs111827785	153775785	C/T	0.414	0.540	Het	3.309	1.744	6.277	0.000229	0.419	0.456	T v C	1.185	0.741	1.895	0.479
SMA	rs762513	153675171	A/G	0.363	0.252	Het	0.397	0.229	0.689	0.000829	0.340	0.362	G v A	1.150	0.695	1.903	0.587
SMA	G6PD202	153764217	G/A	0.205	0.139	Het	0.323	0.160	0.653	0.000847	0.193	0.172	A v G	0.934	0.505	1.730	0.829
RD	G6PD376	153763492	A/G	0.397	0.352	Het	0.306	0.153	0.612	0.000529	0.371	0.458	G v A	1.369	0.748	2.505	0.310
RD	rs762513	153675171	A/G	0.363	0.246	Het	0.309	0.149	0.641	0.000957	0.340	0.320	G v A	0.886	0.471	1.667	0.707
RD	rs12393550	153758660	G/A	0.387	0.352	Het	0.338	0.171	0.668	0.001323	0.352	0.446	A v G	1.315	0.725	2.387	0.369
RD	rs762515	153764528	T/C	0.396	0.356	Het	0.344	0.175	0.676	0.001470	0.365	0.455	C v T	1.296	0.718	2.339	0.390
RD	rs5986990	153761628	G/A	0.395	0.356	Het	0.345	0.175	0.678	0.001503	0.367	0.462	A v G	1.347	0.750	2.420	0.319
RD	rs28470352	153753490	T/A	0.394	0.356	Het	0.354	0.180	0.696	0.002003	0.369	0.455	A v T	1.288	0.714	2.324	0.401
RD	rs2515904	153762771	G/C	0.268	0.212	Het	0.377	0.183	0.774	0.005782	0.233	0.247	C v G	0.944	0.479	1.863	0.868
RD	rs2515905	153762075	G/A	0.269	0.212	Het	0.379	0.185	0.776	0.005878	0.241	0.247	A v G	0.927	0.471	1.824	0.825
Acid.	rs762515	153764528	T/C	0.396	0.327	Het	0.337	0.201	0.566	0.000023	0.365	0.374	C v T	1.112	0.696	1.777	0.657
Acid.	G6PD376	153763492	A/G	0.397	0.335	Het	0.344	0.205	0.579	0.000036	0.371	0.362	G v A	1.024	0.632	1.659	0.923
Acid.	rs28470352	153753490	T/A	0.394	0.330	Het	0.346	0.205	0.584	0.000043	0.369	0.374	A v T	1.101	0.689	1.759	0.687
Acid.	rs5986990	153761628	G/A	0.395	0.331	Het	0.350	0.209	0.587	0.000044	0.367	0.374	A v G	1.105	0.691	1.766	0.677
Acid.	rs12393550	153758660	G/A	0.387	0.328	Het	0.357	0.213	0.599	0.000062	0.352	0.355	A v G	1.039	0.641	1.685	0.877
Acid.	rs762516	153764663	C/T	0.269	0.216	Het	0.373	0.216	0.645	0.000275	0.239	0.224	T v C	1.051	0.617	1.789	0.855
Acid.	rs2515905	153762075	G/A	0.269	0.215	Het	0.383	0.223	0.657	0.000341	0.241	0.223	A v G	1.053	0.617	1.794	0.851
Acid.	rs2515904	153762771	G/C	0.268	0.215	Het	0.384	0.223	0.660	0.000380	0.233	0.223	C v G	1.077	0.630	1.840	0.787
Acid.	rs111827785	153775785	C/T	0.414	0.500	Het	2.507	1.377	4.561	0.002455	0.419	0.486	T v C	1.148	0.728	1.809	0.553

* All models adjusted for age and ethnicity; OR: odds ratio; LCL: lower 95% confidence limit; UCL: upper 95% confidence limit; Het: Heterozygous advantage; Dom: dominance effect; SM: severe malaria; SMA: severe malarial anaemia; RD: respiratory distress; Acid.: acidosis

The correlation between the 29 SNPs was high (linkage disequilibrium D’ median (IQR): all subjects 0.987 (0.811–0.997); female controls 0.988 (0.731–0.998)). Similarly, LD was high across this region in the trio parents (all: 0.998 (0.995–0.999); female only: 0.998 (0.995–0.999)) and children (0.998 (0.996–0.999)) (S2 Fig.). This high LD allowed us to define a small number of haplotypes/G6PD alleles (4) that accounted for 99.6% of all alleles typed for the “core” region (haplotype 1 = GGGAGTC, 2 = AACGGCT (6 mutations), 3 = AACGACT (7 mutations), 4 = AGGGGCC (3 mutations)). Female controls had a higher frequency of the three haplotypes (2–4) containing mutations. Whilst protective effects were observed in females (and not males) for these three haplotypes (OR 0.683–0.783) compared to the common type (haplotype 1, frequency ∼60%), they were not statistically significant (P>0.186), due to the heterozygous nature of the protection in females (S4 Table). Further analysis accounting for the genotypic combinations of *G6PD* alleles confirmed that a combination of haplotypes 1 and either 2 or 3 were protective (OR<0.38, P<0.006) compared to a double haplotype 1 (wild-type) genotype (Table 3). This result shows that haplotypes with the 376G mutation have similar protective effect in heterozygotes irrespective of the presence or absence of the 202A mutation, indicating that the 376G mutation is causal. The genotypic combination of haplotypes 1 and 4 also had a potentially protective effect (OR = 0.599), but it failed to reach statistical significance (P = 0.11).

**Table 3:** Severe malaria and combinations of haplotypes. *

Haplotypes **	G6PD type ***	Control (n = 255)%	Case (n = 236) %	Odds ratio ****	95% LCL	95% UCL	P-value
1,1	B, B	32.2	44.1	1.000	-	-	-
**1,2**	**A, B**	**8.6**	**3.8**	**0.228**	**0.088**	**0.595**	**0.0025**
**1,3**	**A-, B**	**25.9**	**15.7**	**0.376**	**0.215**	**0.657**	**0.0006**
1,4	B, B	14.1	13.6	0.599	0.319	1.122	0.1095
2,2	A, A	0.4	0.8	1.066	0.077	14.789	0.9621
2,3	A-, A	1.6	2.5	1.351	0.297	6.156	0.6974
2,4	A, B	0.8	0.8	1.781	0.215	14.749	0.5926
3,3	A-, A-	3.5	5.1	1.173	0.429	3.207	0.7554
3,4	A-, B	3.1	2.5	0.515	0.150	1.763	0.2905
4,4	B, B	2.7	1.7	0.367	0.092	1.472	0.1574
other	Mixed	7.1	9.3	0.995	0.454	2.183	0.9903

LCL: lower confidence interval, UCL: upper confidence interval

* Based on rs5986990, rs2515905, rs2515904, G6PD376, G6PD202, rs762515, rs762516

** Haplotype 1 = GGGAGTC, 2 = AACGGCT, 3 = AACGACT, 4 = AGGGGCC

*** Based on G6PD202 and G6PD376

**** Adjusted for age and ethnicity

The statistically significant results are bolded.

It is possible that the greater protective effects of haplotypes 2 and 3, could be due to the presence of more mutations (≥6), leading to a possible compound heterozygous advantage effect. The number of heterozygous genotype calls in female controls was greater than in cases (case vs. control median / mean: All SNPs 10 / 9.1 vs. 7 / 7.6, P<0.001; 7 core SNPs, 3 / 3.2 vs. 0 / 2.1, P<0.0001). The *Tajima’s D* metric was applied to assess if the excess number of heterozygous alleles led to evidence of balancing selection in the *G6PD* gene. There was very strong evidence of balancing selection across all groups (*Tajima’s D* > 2.6, female controls 2.9). The magnitude of effect is at the extreme positive tail of an observed negatively centred African population distribution [21], where predominantly negative values demonstrate either slow growth from a small population size, or a bottleneck that is much older than that of non-Africans [21]. This result implies that the (high) allele frequency of the SNPs in the G6PD gene is maintained mainly, and perhaps entirely, by the protection against severe malaria of heterozygous females through a balancing selection mechanism. This selection mechanism is also predicted by population genetic theory [22], and consistent with empirical data from other studies [8,18]. Such mechanisms exist at other malaria candidate loci in the autosomal regions, for example at the HbAS sickle trait [23]. There was no evidence of epistatic effects between HbS and *G6PD* on severe malaria in females (P = 0.34), nor males (P = 0.98). Similarly, no evidence of epistasis between alpha thalassaemia and *G6PD* (female P = 0.44; male P = 0.21).

## Discussion

Although G6PD A- deficiency is known to protect against severe malaria in African populations, the underlying genetic mechanisms are not well understood. *P*. *falciparum* development is hindered in G6PD deficient red cells [24], slowing the rate of parasite replication and reducing the likelihood of severe disease. Suggested mechanisms include more efficient clearance of the infected erythrocytes [25], lower abundance of *P*. *falciparum* 6-phosphogluconolactonase mRNA in parasites from G6PD-deficient children [26], and impaired parasite replication [27]. By using the largest set of *G6PD* (and surrounding loci) SNPs (n = 68) in a genetic association study, within a Tanzanian case-control setting, we have established a set of new *G6PD* alleles associated with protection. These SNPs need to be further investigated to assess their effect on enzyme function in light of potential use of primaquine for malaria elimination. After validation, these SNPs may be used to identify G6PD-deficient individuals in studies of primaquine efficacy.

Further, we have shown that the protective effect of G6PD deficiency is limited to female heterozygotes. This is entirely consistent with heterozygote advantage and balancing selection, relying on alleles at modest frequency and avoiding fixation, where protection provided by this G6PD deficiency against severe malaria is offset by increased risk of life-threatening complications, such as neonatal jaundice and haemolytic crises. In female heterozygotes, random inactivation of one of the two X chromosomes results in some cells with normal enzyme and others with mutant enzyme [11, 28, 29], reducing the risk of both anaemia and severe malaria. We expect that the fitness of normal male hemizygotes is the same as that of normal female homozygotes (since all red cells will contain fully functional enzyme), and population genetic theory also suggests that the fitness of G6PD-deficient male hemizygotes is the same as that of G6PD-deficient female homozygotes. Under these conditions, it is expected that the female heterozygote must be the genotype with the highest fitness [22]. Two independent studies [8, 18] in two different populations, nearly 40 years apart, are consistent in this regard, with G6PD deficiency A− being a balanced polymorphism with heterozygote advantage. Similarly, as the G6PD deficiency A− has been estimated to be at least 5000 years old [3], balancing selection would account for it not having gone to fixation [22]. Further, balancing selection has been observed in autosomal malaria candidate regions like *FREM3*, the major histocompatibility complex, and the sickle cell trait loci [23].

Hitherto, there has been much uncertainty about the relationship between G6PD status and susceptibility to malaria, due in part to G6PD and malaria phenotypic complexity and misclassification, and potentially also from the genetic complexity of the *G6PD* locus with the presence of multiple functional SNPs, each of which may separately modify an individual’s enzyme status and susceptibility to malaria. Until very recently, almost all-large association studies genotyped a limited number of *G6PD* SNPs (e.g. G6PD202 / G6PD376 for A- deficiency), and this approach has been too blunt to capture the full picture. However, analysis of 58 *G6PD* SNPs has demonstrated major G6PD haplotypic differences between sympatric ethnic groups in Mali [9] and genotyping of the G6PD968 polymorphism in addition to 202/376 revealed a female protective in a Gambian population [8]. With hindsight, it is clear that genotyping of G6PD968 in another study in the same population [14] would have prevented misclassification of two-thirds of the G6PD-deficient samples and the erroneous reporting of a male hemizygous protective effect. Other studies reporting male hemizygous protective effects may also be confounded by allelic heterogeneity, which could be avoided by more comprehensive genotyping and by phenotypic testing for G6PD enzyme activity. A comprehensive study would include a full genetic survey of the *G6PD* and surrounding regions, with multiple populations and ethnic groups, leading to a more complete map of *G6PD* that would guide future evolutionary and association studies.

A surprising association result is that the G6PD376 mutation is potentially more influential than G6PD202 and haplotypes that contain the 376G with or without the 202A mutation appear to be similar in terms of protective effect on heterozygotes. The 202A mutation is thought to have a more severe effect on enzyme function than the 376G mutation (∼12% and ∼83% of normal function, respectively [10, 30]) and coexistence of 202A/376G is responsible for G6PD A- enzyme deficiency [20], but it is possible that more subtle changes in enzyme structure or function also affect the outcome of malaria infection. Fully understanding the role of *G6PD* requires further correlation of enzymatic activity with full sequences of *G6PD* and surrounding loci, set within large severe-malaria case and control studies. There have been no such studies to date. A recent study of four *G6PD* deficiency polymorphisms (202, 376, 968, Ilesha) and associated enzymatic activities for 110 sequenced genes in African Americans [31] but included only 54 heterozygous females. Enzymatic activity for G6PD376G (A+, n = 28), 376G/202A (A- deficiency, n = 23), 376G/968C (A-, n = 1), 376G/202A/968C (A-, n = 1) and Ilesha (E156K, Nigeria, non A-, n = 1) alleles was estimated to be ∼83%, ∼53% ∼58%, ∼11% and ∼75% of normal, respectively. These results are consistent with deficiency increasing with additional A- related polymorphism, and by implication will change levels of protection or susceptibility to malaria. Another recent study [32] in 1,828 Kenyan children suggested that G6PD202 was responsible for the majority of G6PD enzyme deficiency but that 376G increases the risk of deficiency in 202AG heterozygotes. Neither study considered malaria outcomes.

In summary, through a much better understanding of the true extent of genetic diversity within and around the *G6PD* locus, we have identified alleles associated with protection from severe malaria in Tanzania, driven by a balancing heterozygous advantage mechanism. Further work should extend the mapping of diversity at this genomic region, and identify how the resulting mutations relate to enzyme function, and how they vary between region and ethnic group. In doing so, genetic epidemiological studies are likely to provide robust and repeatable data, which may be used to develop interventions, and improve malaria disease control.

## Materials and Methods

### Study participants

The study was conducted in the Teule district hospital and surrounding villages in Muheza district, Tanga region. In this region, mortality in children under 5 years of age is 165 per 1000 (Tanzanian census 2002) and transmission of *P*. *falciparum* malaria is intense (50–700 infected bites/person/year) and perennial, with two seasonal peaks [17]. The community prevalence of *P*. *falciparum* parasites in children aged 2–5 years in the study area was recorded as 88.2% in 2002 [17].

Severe malaria cases (n = 506), aged six months to ten years, were recruited during a one-year period between June 2006 and May 2007, with patent parasitaemia, and fulfilling any one of the following eligibility criteria; history of 2 or more convulsions in last 24 hours, prostration (unable to sit unsupported if <9 months of age or drink at any age), reduced consciousness (Blantyre Coma scale<5), respiratory distress, jaundice, severe anaemia (Hemocue Hb < 5g/dL), acidosis (Blood lactate ≥ 5 mmol/L), hypoglycaemia (blood glucose < 2.5mmol/L). Cases were defined as having had cerebral malaria if their Blantyre coma score was less than or equal to 3 on presentation or early during admission. Participants with co-existing severe or chronic medical conditions (e.g. bacterial pneumonia, kwashiorkor) unrelated to a severe malarial infection were excluded. All cases were confirmed as having *P*. *falciparum* malaria parasites. Parasite infection was initially assessed by rapid diagnostic test (HRP-2—Parascreen Pan/Pf) and confirmed by double read Geimsa-stained thick blood films. Residence and ethnic group of both parents was recorded from information provided by the caregiver for each child [17].

Controls (n = 477) were recruited matched on ward of residence, ethnicity and age using household lists during a four-week period in August 2008. Study participants resided in 33 geographical wards (including Mtindiro 9.6%, Kwafungo 8.5%, Mkata 6.3%, Kwedizinga 6.0%, others each < 5.0%) surrounding Muheza town in the Tanga region. The participants had a median age of ∼2.6 years, and were predominantly from seven ethnic groups (see Table 1). Because of limited sample size, we did not perform a detailed analysis based on different ethnic groups or wards of residence.

To complement the case-control collection, we collected samples anonymously from 60 healthy parental and child trios (120 parents, 60 children) during 2007 and 2008 from lowland villages near the West Usambara mountains in the Tanga region of Tanzania, which ranges from high to medium levels of malaria transmission. No malaria phenotypic data is available on these individuals, but their genotypic profiles were used to provide validation data of the genetic aspects of the case-control study.

### Sample collection and preparation

Approximately 3ml of venous blood was collected from participants into EDTA vacutainers. A blood film was prepared and haemoglobin levels measured by hemocue. Children in the control group with haemoglobin levels of <11g/DL were referred to the nearest health facility; those with a positive blood film were treated in line with Tanzanian national treatment guidelines and excluded from the genetic analysis. Samples were spun at 5000rpm for 5 minutes and the plasma removed and stored for future analysis. DNA was extracted and purified from the blood cell pellet using a nucleon kit (see [17] for details).

### Sample genotyping

Genomic DNA samples were genotyped on a Sequenom MassArray genotyping platform [17,33]. The iPlex genotyping assays included 68 G6PD single nucleotide polymorphism (SNP) positions (identified through resequencing and the 1000 genomes project, described in [9, 32]), HbS (rs334), HbC (rs33930165), HbE (rs33950507), and two SNPs that allow an estimate of the ABO blood group rs8176719, rs8176746). In particular, the rs8176719 derived allele results in a non-functional enzyme, and group O individuals are DD, while non-O Individuals are either II or ID. In addition, rs8176746 is involved in the enzyme's substrate selection and therefore defines either the A or B blood groups. A full list of SNPs can be found in S1 Table. The α^3.7^-thalssaemia deletion was typed separately by PCR [17].

### Statistical analysis

All analyses involving SNPs were stratified by gender. Genotypic deviations from Hardy-Weinberg equilibrium (HWE) in females were assessed using a Chi-square statistical test. SNPs were excluded from analysis if they had at least 10% of genotype calls missing, more than 2% of males genotype calls were (falsely) called heterozygous, or if there was a distortion from HWE in female controls (HWE Chi-square P<0.00001) [9]. On this basis, 6 SNPs were excluded (rs766419, rs743545, rs743548, b36_153424319, rs2472393, b36_153426354). A further 33 SNPs with minor allele frequency less than 1% were also removed, leaving 29 high quality SNPs for association analysis (listed in S2 Table). The 29 SNPs are located in a genomic region with known regulatory capacity (transcription factor binding and DNase peaks (regulomedb.org), and both promoter and enhancer histone marks, with a number of different binding proteins and regulatory motif changes (compbio.mit.edu/HaploReg)).

Case-control association analysis using SNP alleles or genotypes was undertaken within a logistic regression framework, and included age and ethnic group as covariates. In this approach we modelled the SNP of interest assuming several related genotypic mechanisms (additive, dominant, recessive, heterozygous advantage and general models) and reported the minimum p-value from these correlated tests. Epistatic effects between polymorphisms were considered by inclusion of statistical interactions in these models. The haplotypes of females were inferred from genotypes using an expectation-maximization algorithm [34]. Haplotype association testing was performed using the regression models [34]. Linkage disequilibrium was estimated using the pairwise *D-prime* (and *R-square*) metrics [35]. Performing multiple statistical tests leads to inflation in the occurrence of false positives. A Bonferroni correction would be too conservative because all SNPs are from the same gene. A permutation approach that accounted for the correlation between tests estimated that a p-value cut-off of 0.006 would ensure a global significance level of 5%. All analyses were performed using the R statistical software. The R *haplo*.*stat* library was used to implement haplotype analysis. The *Tajima’s D* metric was used to quantify evidence of balancing selection based on the allele frequency spectrum [36]. A negative *Tajima's D* indicates purifying selection and/or population size expansion, while positive values may indicate balancing selection. Values greater than +2 or less than -2 are likely to be significant [36].

### Ethics

All DNA samples were collected and genotyped following signed and informed written consent from a parent or guardian. Ethics approval for all procedures was obtained from both LSHTM (#2087) and the Tanzanian National Institute of Medical Research (NIMR/HQ/R.8a/Vol.IX/392).

## Supporting Information

S1 Table List of SNPs. (DOCX)

S2 Table *G6PD*, *IKBKG* and *CTAG1A/B* SNPs and minor allele frequencies (MAF>1%). * with minor allele frequencies (MAF) in excess of 1%** b36_153411172, b36_153412566, b36_153412620, b36_153412734, b36_153412861, b36_153413455, rs72554665, b36_153413799, b36_153414077, b36_153414378, G6PD968, b36_153414531, b36_153414709, b36_153414937, b36_153415014, G6PD680, rs5986875, b36_153415799, rs5030868, rs5030872, b36_153415904, b36_153416019, b36_153416656, b36_153416679, b36_153417405, b36_153417417, b36_153424232, b36_153426313, b36_153427408, b36_153427466, rs5986992, b36_153429686, and rs5986997 had MAF<1% and were excluded from association analysis.
(DOCX)

S3 Table *G6PD*, *IKBKG* and *CTAG1A/B* loci polymorphisms and minor allele frequencies in the child-parental trio study. rs33950507 (HbC)- G, b36_153412566-C, b36_153412620-C, b36_153412734-G, b36_153412861-G, b36_153413455-A, b36_153413678-C, b36_153413799-G, b36_153414378-G, G6PD968-T, b36_153414531-C, b36_153414709-C, rs598699-G, b36_153414937-T, G6PD680-G, 36_153415799-G, b36_153415828-G, G6PD542-A, b36_153415904-C, b36_153416019-C, b36_153416656-G, b36_153416679-A, b36_153417405-A, b36_153417417-A, b36_153424232-T, b36_153426313-G, b36_153427466-T, rs5986992-C, b36_153429686-G, rs5986997-C are all fixed; b36_153411172-G, b36_153415014-A, rs5986875-A, and b36_153426354-C all had allele frequencies less than 1%.
(DOCX)

S4 Table Haplotype analysis. * rs5986990, rs2515905, rs2515904, G6PD376, G6PD202, rs762515, rs762516; LCL lower confidence interval, UCL upper confidence interval
(DOCX)

S1 Fig Association results for sub-clinical severe malaria phenotypes (males—solid, females—hollow circles). The horizontal dashed lines represent a p-value cut-off of 0.006. Some SNP results are not presented because of statistical model non-convergence due to low numbers of cases and low minor allele frequency.
(DOCX)

S2 Fig Pairwise linkage disequilibrium. (Top left *D’*, Bottom right *R-square*; black = 0 -> white = 1)
(a) All cases and controls
(b) Female controls
(c) All parents in the Trio study
(d) Female parents in the Trio study
(DOCX)
