# Supplementary material for: African Glucose-6-Phosphate Dehydrogenase Alleles Associated with Protection from Severe Malaria in Heterozygous Females in Tanzania
Source: PLoS Genet. 2015 Feb 11;11(2):e1004960. doi: 10.1371/journal.pgen.1004960 (PMC4335500; doi:10.1371/journal.pgen.1004960)
Supplement: S1 Table — (DOCX) [file pgen.1004960.s001.docx]

**S1 Table**

**List of SNPs**

| Chr | Position | RS number | Alternative names | Gene | Reference |
| --- | --- | --- | --- | --- | --- |
| 9 | 136131322 | rs8176746 | - | *ABO* | C |
| 9 | 136132909 | rs8176719 | - | *ABO* | I |
| 11 | 5248173 | rs33950507 |  | *HBB* | G |
| 11 | 5248232 | rs334 | HbS | *HBB* | A |
| 11 | 5248233 | rs33930165 |  | *HBB* | G |
| X | 153554404 | rs766420 |  | *G6PD* | C |
| X | 153554661 | rs766419 |  | *G6PD* | A |
| X | 153593150 | rs743545 |  | *G6PD* | G |
| X | 153626649 | rs915941 |  | *G6PD* | C |
| X | 153626738 | rs915942 |  | *G6PD* | G |
| X | 153650420 | rs743548 |  | *G6PD* | T |
| X | 153675171 | rs762513 |  | *G6PD* | A |
| X | 153753490 | rs28470352 |  | *G6PD* | T |
| X | 153755336 | rs61042368 |  | *G6PD* | G |
| X | 153757734 | rs12389569 |  | *G6PD* | G |
| X | 153757978 | b36_153411172 |  | *G6PD* | C |
| X | 153758660 | rs12393550 |  | *G6PD* | G |
| X | 153759372 | b36_153412566 |  | *G6PD* | C |
| X | 153759426 | b36_153412620 |  | *G6PD* | C |
| X | 153759540 | b36_153412734 |  | *G6PD* | G |
| X | 153759667 | b36_153412861 |  | *G6PD* | G |
| X | 153760261 | b36_153413455 |  | *G6PD* | A |
| X | 153760429 | b36_153413623 |  | *G6PD* | G |
| X | 153760484 | rs72554665 |  | *G6PD* | C |
| X | 153760508 | rs2071429 |  | *G6PD* | G |
| X | 153760605 | b36_153413799 |  | *G6PD* | G |
| X | 153760654 | rs2230037 |  | *G6PD* | G |
| X | 153760883 | b36_153414077 | Bari/P396A | *G6PD* | G |
| X | 153760953 | rs2230036 |  | *G6PD* | C |
| X | 153761184 | b36_153414378 |  | *G6PD* | G |
| X | 153761240 | rs76723693 | G6PD968/L323P | *G6PD* | T |
| X | 153761337 | b36_153414531 |  | *G6PD* | C |
| X | 153761515 | b36_153414709 |  | *G6PD* | C |
| X | 153761564 | rs73573478 |  | *G6PD* | G |
| X | 153761628 | rs5986990 |  | *G6PD* | G |
| X | 153761743 | b36_153414937 |  | *G6PD* | T |
| X | 153761820 | b36_153415014 |  | *G6PD* | T |
| X | 153762075 | rs2515905 |  | *G6PD* | G |
| X | 153762340 | rs137852328 | G6PD680/D227V | *G6PD* | G |
| X | 153762392 | rs5986875 |  | *G6PD* | G |
| X | 153762605 | b36_153415799 |  | *G6PD* | G |
| X | 153762634 | rs5030868 |  | *G6PD* | G |
| X | 153762655 | rs5030872 | G6PD542/D181V | *G6PD* | A |
| X | 153762710 | b36_153415904 |  | *G6PD* | C |
| X | 153762771 | rs2515904 |  | *G6PD* | G |
| X | 153762825 | b36_153416019 |  | *G6PD* | C |
| X | 153763462 | b36_153416656 |  | *G6PD* | G |
| X | 153763485 | b36_153416679 |  | *G6PD* | A |
| X | 153763492 | rs1050829 | G6PD376/ N126D | *G6PD* | A |
| X | 153764211 | b36_153417405 |  | *G6PD* | A |
| X | 153764217 | rs1050828 | G6PD202/V98M | *G6PD* | G |
| X | 153764223 | b36_153417417 |  | *G6PD* | A |
| X | 153764528 | rs762515 |  | *G6PD* | T |
| X | 153764663 | rs762516 |  | *G6PD* | C |
| X | 153769889 | rs73641103 |  | *G6PD/IKBKG* | G |
| X | 153771038 | b36_153424232 |  | *G6PD/IKBKG* | T |
| X | 153771125 | rs73573488 |  | *G6PD/IKBKG* | A |
| X | 153771296 | rs2472393 |  | *G6PD/IKBKG* | T |
| X | 153773062 | b36_153426256 |  | *G6PD/IKBKG* | C |
| X | 153773119 | b36_153426313 |  | *G6PD/IKBKG* | G |
| X | 153773160 | b36_153426354 |  | *G6PD/IKBKG* | A |
| X | 153773526 | b36_153426720 |  | *G6PD/IKBKG* | A |
| X | 153774214 | b36_153427408 |  | *G6PD/IKBKG* | T |
| X | 153774272 | b36_153427466 |  | *G6PD/IKBKG* | T |
| X | 153775785 | rs111827785 |  | *G6PD/IKBKG* | C |
| X | 153776107 | rs5986992 |  | *G6PD/IKBKG* | C |
| X | 153776492 | b36_153429686 |  | *G6PD/IKBKG* | G |
| X | 153827549 | rs5986997 |  | *CTAG1A/B* | C |
| X | 153827637 | rs4898389 |  | *CTAG1A/B* | G |
| X | 153828269 | rs5986877 |  | *CTAG1A/B* | G |
| X | 153829693 | rs7879049 |  | *CTAG1A/B* | A |
| X | 153834100 | rs7053878 |  | *CTAG1A/B* | T |
| X | 153836171 | rs60030796 |  | *CTAG1A/B* | A |
