# Supplementary material for: African Glucose-6-Phosphate Dehydrogenase Alleles Associated with Protection from Severe Malaria in Heterozygous Females in Tanzania
Source: PLoS Genet. 2015 Feb 11;11(2):e1004960. doi: 10.1371/journal.pgen.1004960 (PMC4335500; doi:10.1371/journal.pgen.1004960)
Supplement: S2 Table — * with minor allele frequencies (MAF) in excess of 1%** b36_153411172, b36_153412566, b36_153412620, b36_153412734, b36_153412861, b36_153413455, rs72554665, b36_153413799, b36_153414077, b36_153414378, G6PD968, b36_153414531, b36_153414709, b36_153414937, b36_153415014, G6PD680, rs5986875, b36_153415799, rs5030868, rs5030872, b36_153415904, b36_153416019, b36_153416656, b36_153416679, b36_153417405, b36_153417417, b36_153424232, b36_153426313, b36_153427408, b36_153427466, rs5986992, b36_153429686, and rs5986997 had MAF<1% and were excluded from association analysis. (DOCX) [file pgen.1004960.s002.docx]

**S2 Table**

***G6PD, IKBKG* and *CTAG1A/B* SNPs and minor allele frequencies (MAF>1%)**

| SNP* | Position | Major/  Minor  alleles | Female control  (n=255) | Male  Control  (n=222) | Female  Case  (n=236) | Male  Case  (n=270) |
| --- | --- | --- | --- | --- | --- | --- |
| rs766420 | 153554404 | G/C | 0.359 | 0.361 | 0.382 | 0.386 |
| rs915941 | 153626649 | A/C | 0.496 | 0.489 | 0.504 | 0.476 |
| rs915942 | 153626738 | G/A | 0.368 | 0.365 | 0.372 | 0.354 |
| rs762513 | 153675171 | A/G | 0.363 | 0.340 | 0.310 | 0.307 |
| rs28470352 | 153753490 | T/A | 0.394 | 0.369 | 0.334 | 0.370 |
| rs61042368 | 153755336 | G/A | 0.154 | 0.160 | 0.152 | 0.142 |
| rs12389569 | 153757734 | G/A | 0.061 | 0.065 | 0.057 | 0.079 |
| rs12393550 | 153758660 | G/A | 0.387 | 0.353 | 0.333 | 0.347 |
| b36_153413623 | 153760429 | G/A | 0.090 | 0.063 | 0.089 | 0.067 |
| rs2071429 | 153760508 | G/A | 0.067 | 0.080 | 0.072 | 0.049 |
| rs2230037 | 153760654 | G/A | 0.237 | 0.241 | 0.288 | 0.320 |
| rs2230036 | 153760953 | C/T | 0.152 | 0.160 | 0.147 | 0.138 |
| rs73573478 | 153761564 | G/A | 0.154 | 0.160 | 0.144 | 0.133 |
| rs5986990 | 153761628 | G/A | 0.395 | 0.368 | 0.335 | 0.374 |
| rs2515905 | 153762075 | G/A | 0.269 | 0.241 | 0.221 | 0.228 |
| rs2515904 | 153762771 | G/C | 0.268 | 0.233 | 0.221 | 0.227 |
| G6PD376 | 153763492 | A/G | 0.397 | 0.371 | 0.340 | 0.374 |
| G6PD202 | 153764217 | G/A | 0.205 | 0.193 | 0.174 | 0.154 |
| rs762515 | 153764528 | T/C | 0.396 | 0.366 | 0.333 | 0.369 |
| rs762516 | 153764663 | C/T | 0.269 | 0.239 | 0.217 | 0.233 |
| rs73641103 | 153769889 | G/A | 0.026 | 0.014 | 0.021 | 0.017 |
| b36_153426256 | 153773062 | C/T | 0.146 | 0.161 | 0.155 | 0.142 |
| b36_153426720 | 153773526 | A/G | 0.010 | 0.014 | 0.011 | 0.013 |
| rs111827785 | 153775785 | C/T | 0.414 | 0.420 | 0.476 | 0.460 |
| rs4898389 | 153827637 | G/A | 0.053 | 0.077 | 0.060 | 0.028 |
| rs5986877 | 153828269 | G/C | 0.067 | 0.084 | 0.074 | 0.048 |
| rs7879049 | 153829693 | A/G | 0.338 | 0.351 | 0.335 | 0.375 |
| rs7053878 | 153834100 | T/A | 0.053 | 0.052 | 0.052 | 0.046 |
| rs60030796 | 153836171 | A/G | 0.051 | 0.067 | 0.032 | 0.043 |

* with minor allele frequencies (MAF) in excess of 1%

** b36_153411172, b36_153412566, b36_153412620, b36_153412734, b36_153412861, b36_153413455, rs72554665, b36_153413799, b36_153414077, b36_153414378, G6PD968, b36_153414531, b36_153414709, b36_153414937, b36_153415014, G6PD680, rs5986875, b36_153415799, rs5030868, rs5030872, b36_153415904, b36_153416019, b36_153416656, b36_153416679, b36_153417405, b36_153417417, b36_153424232, b36_153426313, b36_153427408, b36_153427466, rs5986992, b36_153429686, and rs5986997 had MAF<1% and were excluded from association analysis.
