# Supplementary material for: African Glucose-6-Phosphate Dehydrogenase Alleles Associated with Protection from Severe Malaria in Heterozygous Females in Tanzania
Source: PLoS Genet. 2015 Feb 11;11(2):e1004960. doi: 10.1371/journal.pgen.1004960 (PMC4335500; doi:10.1371/journal.pgen.1004960)
Supplement: S3 Table — rs33950507 (HbC)- G, b36_153412566-C, b36_153412620-C, b36_153412734-G, b36_153412861-G, b36_153413455-A, b36_153413678-C, b36_153413799-G, b36_153414378-G, G6PD968-T, b36_153414531-C, b36_153414709-C, rs598699-G, b36_153414937-T, G6PD680-G, 36_153415799-G, b36_153415828-G, G6PD542-A, b36_153415904-C, b36_153416019-C, b36_153416656-G, b36_153416679-A, b36_153417405-A, b36_153417417-A, b36_153424232-T, b36_153426313-G, b36_153427466-T, rs5986992-C, b36_153429686-G, rs5986997-C are all fixed; b36_153411172-G, b36_153415014-A, rs5986875-A, and b36_153426354-C all had allele frequencies less than 1%. (DOCX) [file pgen.1004960.s003.docx]

**S3 Table**

***G6PD, IKBKG* and *CTAG1A/B* loci polymorphisms and minor allele frequencies in the child-parental trio study**

| SNP | Major Allele | Minor Allele | Parent female  (n=60) | Parent male  (n=60) | Child female  (n=31) | Child male  (n=29) |
| --- | --- | --- | --- | --- | --- | --- |
| rs28470352 | T | A | 0.246 | 0.383 | 0.224 | 0.286 |
| rs61042368 | G | A | 0.164 | 0.148 | 0.138 | 0.214 |
| rs12389569 | G | A | 0.018 | 0.017 | 0.017 | 0.034 |
| rs12393550 | G | A | 0.241 | 0.361 | 0.224 | 0.276 |
| b36_153413623 | G | A | 0.086 | 0.115 | 0.103 | 0.138 |
| rs2071429 | G | A | 0.129 | 0.082 | 0.190 | 0.103 |
| rs2230037 | G | A | 0.319 | 0.262 | 0.293 | 0.172 |
| rs2230036 | C | T | 0.143 | 0.150 | 0.121 | 0.179 |
| b36_153414758 | G | A | 0.155 | 0.148 | 0.121 | 0.207 |
| rs2515905 | G | A | 0.172 | 0.246 | 0.172 | 0.207 |
| rs2515904 | G | C | 0.172 | 0.246 | 0.172 | 0.207 |
| G6PD376 | A | G | 0.246 | 0.389 | 0.224 | 0.259 |
| G6PD202 | G | A | 0.121 | 0.213 | 0.121 | 0.172 |
| rs762515 | T | C | 0.250 | 0.377 | 0.214 | 0.276 |
| rs762516 | C | T | 0.172 | 0.246 | 0.172 | 0.207 |
| b36_153423083 | G | A | 0.043 | 0.066 | 0.034 | 0.034 |
| b36_153424319 | A | G | 0.250 | 0.333 | 0.185 | 0.259 |
| rs2472393 | T | C | 0.129 | 0.082 | 0.190 | 0.103 |
| b36_153426256 | C | T | 0.164 | 0.148 | 0.138 | 0.207 |
| b36_153426720 | A | G | 0.009 | 0.000 | 0.000 | 0.034 |
| b36_153428979 | T | C | 0.448 | 0.459 | 0.411 | 0.448 |
| rs4898389 | G | A | 0.086 | 0.066 | 0.138 | 0.069 |
| rs5986877 | G | C | 0.129 | 0.083 | 0.190 | 0.071 |
| rs7879049 | A | G | 0.362 | 0.350 | 0.310 | 0.241 |
| rs7053878 | T | A | 0.095 | 0.034 | 0.103 | 0.069 |
| rs60030796 | A | G | 0.060 | 0.098 | 0.034 | 0.034 |

rs33950507 (HbC)- G, b36_153412566-C, b36_153412620-C, b36_153412734-G, b36_153412861-G, b36_153413455-A, b36_153413678-C, b36_153413799-G, b36_153414378-G, G6PD968-T, b36_153414531-C, b36_153414709-C, rs598699-G, b36_153414937-T, G6PD680-G, 36_153415799-G, b36_153415828-G, G6PD542-A, b36_153415904-C, b36_153416019-C, b36_153416656-G, b36_153416679-A, b36_153417405-A, b36_153417417-A, b36_153424232-T, b36_153426313-G, b36_153427466-T, rs5986992-C, b36_153429686-G, rs5986997-C are all fixed; b36_153411172-G, b36_153415014-A, rs5986875-A, and b36_153426354-C all had allele frequencies less than 1%.
