# Supplementary material for: African Glucose-6-Phosphate Dehydrogenase Alleles Associated with Protection from Severe Malaria in Heterozygous Females in Tanzania
Source: PLoS Genet. 2015 Feb 11;11(2):e1004960. doi: 10.1371/journal.pgen.1004960 (PMC4335500; doi:10.1371/journal.pgen.1004960)
Supplement: S4 Table — * rs5986990, rs2515905, rs2515904, G6PD376, G6PD202, rs762515, rs762516; LCL lower confidence interval, UCL upper confidence interval (DOCX) [file pgen.1004960.s004.docx]

**S4 Table**

**Haplotype Analysis**

| Haplotype* | Female  Control | Female  Case | Female  Odds ratio | Female 95% LCL | Female UCL | Female P-value | Male Control | Male Case | Male Odds ratio | Male  95% LCL | Male 95% UCL | Male P-value |
| --- | --- | --- | --- | --- | --- | --- | --- | --- | --- | --- | --- | --- |
| GGGAGTC | 0.603 | 0.666 | 1.000 |  |  |  | 0.628 | 0.626 | 1.000 |  |  |  |
| AACGGCT | 0.063 | 0.049 | 0.683 | 0.367 | 1.273 | 0.231 | 0.043 | 0.077 | 1.336 | 0.872 | 2.046 | 0.183 |
| AACGACT | 0.205 | 0.173 | 0.783 | 0.546 | 1.125 | 0.186 | 0.196 | 0.151 | 0.919 | 0.710 | 1.189 | 0.520 |
| AGGGGCC | 0.125 | 0.113 | 0.789 | 0.514 | 1.210 | 0.278 | 0.124 | 0.143 | 1.065 | 0.797 | 1.423 | 0.670 |

* rs5986990, rs2515905, rs2515904, G6PD376, G6PD202, rs762515, rs762516; LCL lower confidence interval, UCL upper confidence interval
