# Supplementary material for: African Glucose-6-Phosphate Dehydrogenase Alleles Associated with Protection from Severe Malaria in Heterozygous Females in Tanzania
Source: PLoS Genet. 2015 Feb 11;11(2):e1004960. doi: 10.1371/journal.pgen.1004960 (PMC4335500; doi:10.1371/journal.pgen.1004960)
Supplement: S1 Fig — The horizontal dashed lines represent a p-value cut-off of 0.006. Some SNP results are not presented because of statistical model non-convergence due to low numbers of cases and low minor allele frequency. (DOCX) [file pgen.1004960.s005.docx]

**S1 Figure**

**Association results for sub-clinical severe malaria phenotypes (males - solid, females – hollow circles). The horizontal dashed lines represent a p-value cut-off of 0.006. Some SNP results are not presented because of statistical model non-convergence due to low numbers of cases and low minor allele frequency.**

**
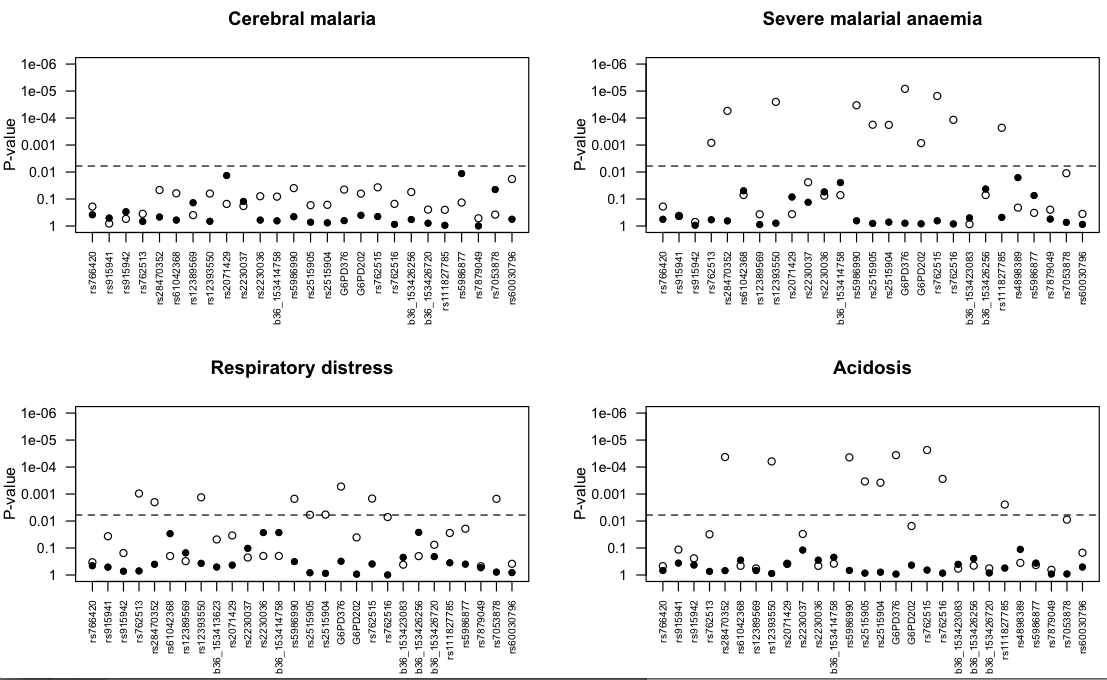
**
