# Supplementary material for: African Glucose-6-Phosphate Dehydrogenase Alleles Associated with Protection from Severe Malaria in Heterozygous Females in Tanzania
Source: PLoS Genet. 2015 Feb 11;11(2):e1004960. doi: 10.1371/journal.pgen.1004960 (PMC4335500; doi:10.1371/journal.pgen.1004960)

**S2 Figure**

**Pairwise linkage disequilibrium**

**(Top left *D’*, Bottom right *R-square*; black = 0 -> white = 1)**

**(a) All cases and controls**

**
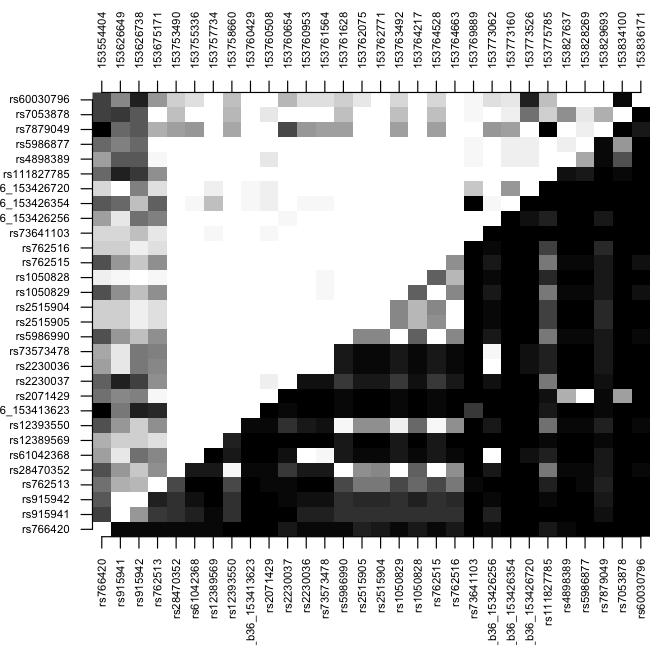
**

**(b) Female controls**

**
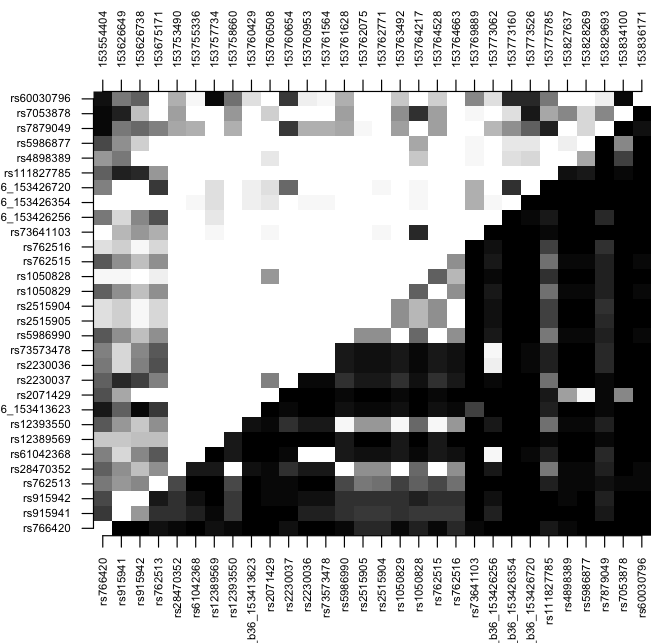
**

**(c) All parents in the Trio study**

**
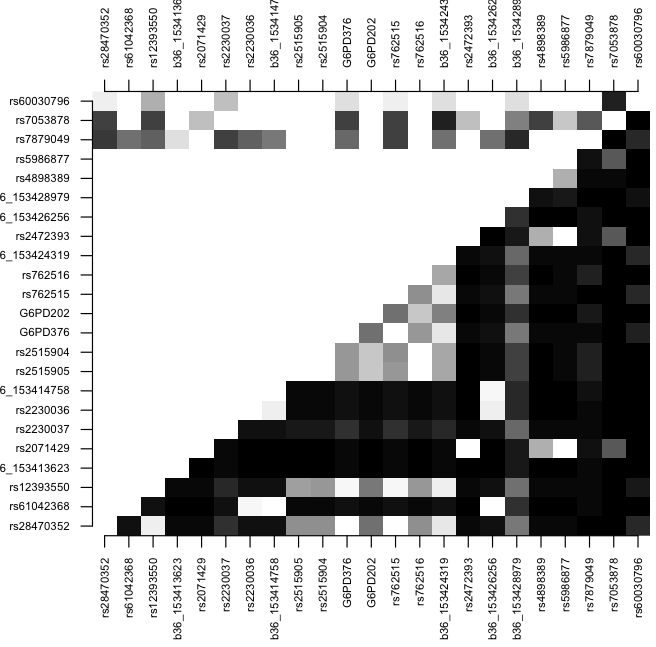
**

**(d) Female parents in the Trio study**


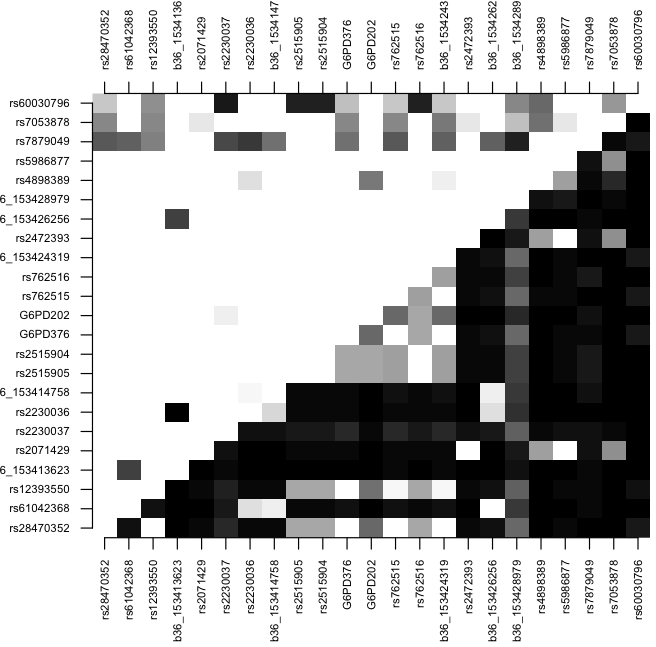

Supplement: S2 Fig — (Top left D’, Bottom right R-square; black = 0 -> white = 1) (a) All cases and controls (b) Female controls (c) All parents in the Trio study (d) Female parents in the Trio study (DOCX) [file pgen.1004960.s006.docx]
